# Note on the after Treatment of Scarlet Fever by Scalded Oatmeal

**Published:** 1884-12

**Authors:** George Smith

**Affiliations:** Axbridge, Somerset


### NOTE ON THE AFTER TREATMENT OF SCARLET FEVER BY SCALDED OATMEAL.

By GEORGE SMITH, M.R.C.S., Axbridge, Somerset.

As the heading of this Note implies, it is intended here to treat of the subject of the desquamation which follows every case of scarlet fever, however slight, both in regard to its bearings on the patient himself and also those with whom any cast-off particles may come into contact.

Take first the process of desquamation. This, as we all know, varies very much in different individuals, and sometimes it is done by particles so fine as to be hardly perceptible; and these are, I think, a very frequent and most certain source of contagion, by means of clothes and otherwise—much more so indeed than the scales as ordinarily thrown off; and I may here state that it is within my own knowledge that the contagion has been thus carried from one house to another, more than a hundred miles apart, at the end of at least a year from the attack.

Now, to obviate this danger, I have for several years been in the habit of having my patients sponged over the whole surface of their bodies twice a day—commencing, as a rule, about a week from the appearance of the eruption, and continuing the process until desquamation is complete—with a mixture of one ounce of oatmeal to one pint of boiling water; the solution to be made fresh every day and used tepid, or at such a temperature as may be comfortably borne by the back of a finger.

My reason for using this particular combination is, that the gluten in it sticks the scales to each other and to the surface of the body, thus allowing of their being removed, from one sponging to another, without the ordinary risk of infecting either atmosphere or clothes, and greatly lessening the risk of spreading the disease.

Secondly, this same gluten fills up the cracks of the new skin and protects it from cold as, patch after patch, it becomes bare, and thus, to say the least, greatly lessens the risk of the dropsy which so often follows upon this disease.

---

